# Contrasting Microbial Community Assembly Hypotheses: A Reconciling Tale from the Río Tinto

**DOI:** 10.1371/journal.pone.0003853

**Published:** 2008-12-04

**Authors:** Carmen Palacios, Erik Zettler, Ricardo Amils, Linda Amaral-Zettler

**Affiliations:** 1 The Josephine Bay Paul Center for Comparative Molecular Biology and Evolution, Marine Biological Laboratory, Woods Hole, Massachusetts, United States of America; 2 Marine Biological Laboratory NASA Astrobiology Institute, Marine Biological Laboratory, Woods Hole, Massachusetts, United States of America; 3 Centro de Biología Molecular, Universidad Autónoma de Madrid, Madrid, Spain; 4 Sea Education Association, Woods Hole, Massachusetts, United States of America; 5 Centro de Astrobiología, INTA-CSIC, Torrejón de Ardoz, Spain; Centre for DNA Fingerprinting and Diagnostics, India

## Abstract

**Background:**

The Río Tinto (RT) is distinguished from other acid mine drainage systems by its natural and ancient origins. Microbial life from all three domains flourishes in this ecosystem, but bacteria dominate metabolic processes that perpetuate environmental extremes. While the patchy geochemistry of the RT likely influences the dynamics of bacterial populations, demonstrating which environmental variables shape microbial diversity and unveiling the mechanisms underlying observed patterns, remain major challenges in microbial ecology whose answers rely upon detailed assessments of community structures coupled with fine-scale measurements of physico-chemical parameters.

**Methodology/Principal Findings:**

By using high-throughput environmental tag sequencing we achieved saturation of richness estimators for the first time in the RT. We found that environmental factors dictate the distribution of the most abundant taxa in this system, but stochastic niche differentiation processes, such as mutation and dispersal, also contribute to observed diversity patterns.

**Conclusions/Significance:**

We predict that studies providing clues to the evolutionary and ecological processes underlying microbial distributions will reconcile the ongoing debate between the Baas Becking vs. Hubbell community assembly hypotheses.

## Introduction

**Figure 1:**
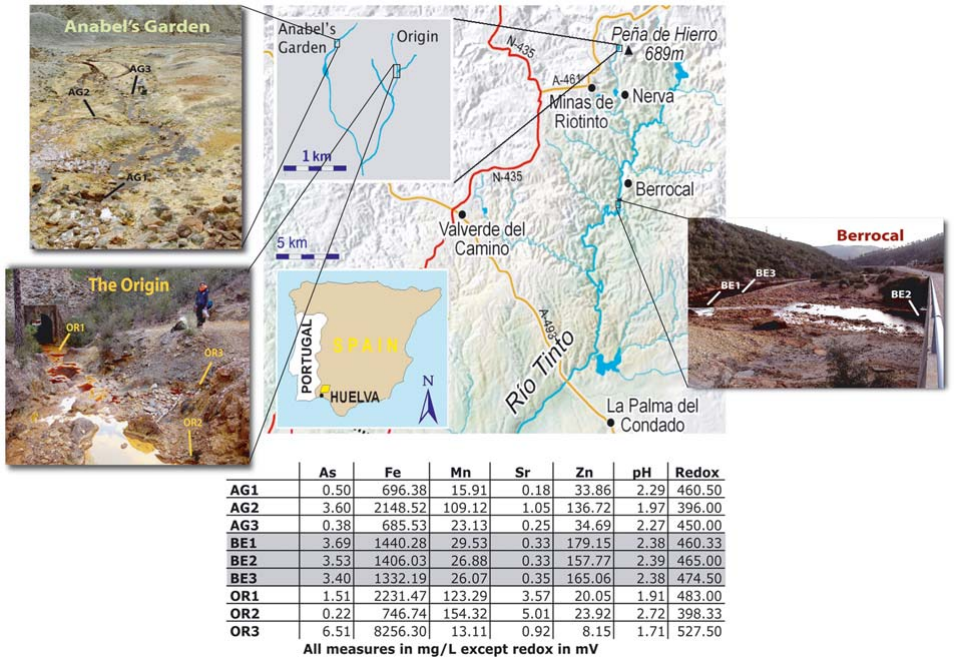
Sampling stations at Rio Tinto: geographic locations and main physico-chemical parameters. A map depicting the geographic location of the Río Tinto in southwestern Spain with insets of our three sampling stations: Anabel's Garden (AG), Origin (OR) and Berrocal (BE). Labeled on the photographs are the relative locations of the three sites sampled for each station. The inset table indicates the physico-chemical parameters that best explained the microbial diversity observed in our study.

Geological and geochemical studies show the Río Tinto to be an acidic river situated at the core of the largest Pyritic Belt on Earth (Fig. 1) whose chemistry has been shaped by the metabolism of chemolithotrophic microbes bioleaching its rich metallic ores for the past 60 My [1]. These microbial activities produce sulfuric acid resulting in a pH below 3 and high concentrations of heavy metals very much like acid mine drainage systems but of natural and very ancient origin. The RT has also attracted the interests of Astrobiologists because its geochemical characteristics are relevant to Martian hematite sites [1]. Research over the past 15 years shows the river contains predominantly microscopic organisms from the three domains of life. Bacteria outnumber archaea by at least ten fold [2]. Eukaryotes are conspicuous and diverse [3] and phototrophs and fungi comprise the largest biomass [4]. While the patchy geochemistry of the RT likely influences the dynamics of the most abundant bacterial populations [2], [5], demonstrating how environmental factors shape microbial community structure of low, moderate and high abundance microbes remains a first order question in microbial ecology research. Environmental tag sequencing methods [6] are ideal for addressing this issue as they allow for deeper sampling of the molecular populations of PCR amplicons. These methods capitalize on the intrinsic phylogenetic information contained in genetically hypervariable regions of the 16S ribosomal RNA gene (rDNA) to simultaneously provide accurate assessments of the relative abundances of all microbial community members and their taxonomic affinities (Text S1). We applied Serial Analysis of Ribosomal Sequence Tags of the V6 hypervariable region (SARST-V6 [7]) to replicate samples from three sites at three stations along the RT (Fig. 1). We coupled these data with measurements of physico-chemical parameters to explore how the environment shapes bacterial community structure. In this study rather than describing the microbial community of the RT, we concentrate on microbial (alpha and beta) ecological diversity. We first aimed to demonstrate that in spite of the dearth of saturation and replication in microbial ecology studies so far, they are in fact essential to provide a comprehensive view of natural microbial assemblages. Our second aim was to cluster short tag sequences into ecologically differentiated populations to shed light on the evolutionary ecological processes underlying microbial diversity patterns in the RT.

## Results and Discussion

### Defining a criterion for clustering sequences in microbial ecology

**Figure 2:**
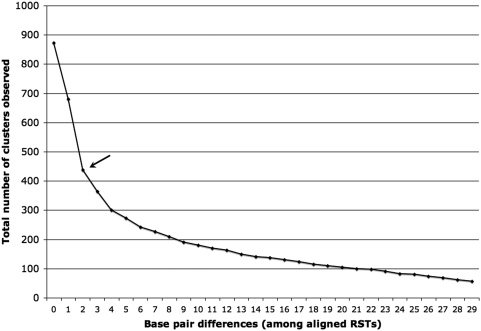
Clusterer output: Number of clusters observed at different cut-off values. Total number of clusters observed as a function of the number of base pair differences between aligned sequences within each cluster. The arrow points to where most microdiversity concentrates (see text for details).

Clustering sequences into operational taxonomic units (OTUs) is the first step in a molecular study exploring ecological diversity. Microbiologists traditionally use a 97% similarity cut-off value to form OTUs that delineate microbial species [8]. Cohan [9] and Polz *et al.* [10] recommend an infraspecific taxonomic level to define significant units in microbial ecology and advocate for an evolutionary ecological criterion to identify distinct microbial populations adapted to a given habitat (ecotypes). Recent bacterial diversity studies identified the presence of microdiverse rDNA clusters at the 99% similarity level denoting bacterial populations that probably arose by selective sweeps followed by effectively neutral diversification [11]–[13]. Furthermore, at least for *Vibrio* spp., these clusters constituted individuals different at the genomic level but whose divergence should be neutral (i.e. with no selective advantage) because of the small spatial scale in which they coexisted [14]. Through environmental sequencing of RT samples we found a total of 1,212 unique ribosomal sequence tags (RSTs) out of 10,529 SARST-V6 tags. RSTs have been deposited in GenBank under accession numbers FJ005322-FJ006533. Most of the microdiversity we observed involved sequences that cluster at >98.5% similarity. The average tag length was 62 bp but the aligned V6 tag regions spanned 142 bp so this represents a 2 bp difference between aligned sequences. The number of clusters at this cut-off was 50% of the maximum possible number of clusters (Fig. 2). Clustering at a 3 bp difference (98%) only decreased the number of clusters by 8.6% (Fig 2). Until the implementation of more appropriate methods than similarity cut-off criteria for defining ecotypes [9], clustering sequences at a 99% similarity for rDNA is the best compromise to form cohesive neutral units of diversity. Linking physicochemical parameters with the resulting genotypic microclusters, however, is still necessary to corroborate that they are differentiated populations that constitute ecologically significant units or ecotypes [9], [10] rather than interoperon heterogeneity within one cell [15].

### In-depth microbial community composition: the known, the new and the rare

**Figure 3:**
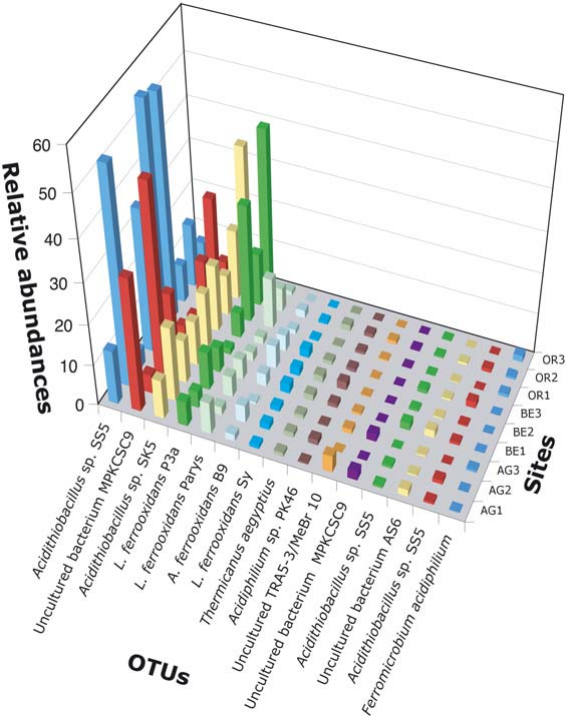
Relative abundances of dominant OTUs at Río Tinto study sites. Histogram of relative abundances of the dominant OTUs (those with more than 40 RSTs) of RT studied sites. (*Acidithiobacillus* sp. SS5 = clone SS5 AY960978.1; SK5 = clone SK5 AY960977.1; *A. ferroxidans* B9 = strain B9 AJ879997.1; MPKCSC9 = clone MPKCSC9 AY766004.1; *L.* = *Leptospirillum*; P3a = strain P3a AF356837.1; Parys = strain Parys AF356838.1; Sy = strain Sy AF356839.1; *Thermicanus aegyptius* = strain ET-5b AJ242495.1; PK46 = AY765995.1; Uncultured TRA5-3/MeBr10 = Uncultured Eubacterium clone TRA5-3 AF047645.1 or clone MeBr10 AY439196.1; AS6 = AF543496.1; *F. acidiphilium* = *Ferromicrobium acidiphilium* AF251436.1). For site names see Fig. 1.

**Figure 4:**
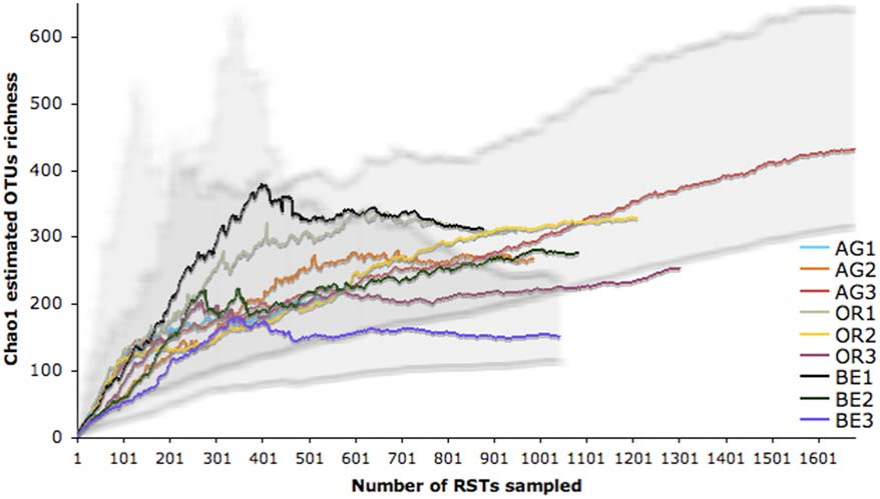
Alpha diversity at Río Tinto study sites. Non-parametric richness estimator Chao1 variation with sampling effort for RT sites. Shadowed areas are 95% confidence intervals of the highest and the lowest richness samples showing overlap of all sites in their estimated OTU richness for the same sampling effort. For site names see Fig 1.

The majority of the 458 OTUs from this study matched sequences previously found in anthropogenically impacted acidic soils or streams but were not previously detected in the RT using culture dependent and other culture independent methods to study microbial diversity. A relatively small number of OTUs dominated all sites (Fig. 3). This pattern is applicable in situations where one or a few factors dominate the ecology of an assemblage [16], as in the RT [2]. The most abundant OTUs previously detected in the RT gave a 100% match to phylogenetic ribotypes of *Acidithiobacillus ferrooxidans* and *Leptospirillum ferrooxidans* and other relatively less abundant ribotypes (Fig. 3) found with the same prevalence, at the same sites during the same time of year by colleagues using DGGE and FISH methods [see Fig. 3 and 4 and Table 5 in ref [2]. The equivalent sites are as follows (this study/ Gonzalez-Toril et al. [2]): (OR1/RT5; OR2/RT2; OR3/RT1; AG/RT6; BE/RT9)]. We interpret this observation as evidence that the same bacterial populations reoccur at certain geochemically stable RT locations. We found *Acidiphilium* sp. related tags to be in lower numbers than in the Gonzalez *et al.* study [2] and attribute this difference to a mismatch in our SARST-V6 primer. Nevertheless, because the bias is consistent across samples it should not invalidate our down-stream ecological diversity analyses [17]. Taxa that had escaped detection in this river so far include the second most common OTU in our dataset (1,654 tags), which matches uncultured bacterial clones MPKCSC9 and TrefC11 (Fig. 3) with 100% similarity. These bacteria dominate macroscopic biofilms thriving in two acidic, metal-rich streams from copper and pyrite mines of Wales and are described as novel acidophilic autotrophic iron oxidizers [18] 99.9% similar to uncultured bacterial clones TRA3-20 and Tui3-12 from acid mine drainage areas in California and New Zealand, respectively. Our OTU also matches these two clones at 100% similarity and has its highest relative abundance at RT sites Anabel's Garden AG1 (175 tags of 539 tags sequenced for this site) and AG3 (783 tags of 1679 tags sequenced), both with similar concentrations of As [19], Fe, S, Zn and pH to the mines where the macroscopic biofilms dominate [18]. Only a few abundant OTUs detected by SARST-V6 (Fig. 3) exhibit low similarity to anything in the databases. In contrast, of the total number of RSTs, 15% differ more than 10% from anything in the databases, and all of them are found at relatively low abundance. This result coincides with previous findings of a “rare biosphere” accompanying the most abundant taxa in microbial communities [20]. Equally remarkable is that a large proportion of less abundant members of the bacterial assemblages in the RT have a 97% similarity to rRNA gene sequences deposited in GenBank whose best match is a sequence of a microbe from an acidic environment. Among these rarer members are bacterial endosymbionts of acidophilic eukaryotes or bacteria previously reported from digestive systems, as well as free-living bacteria observed in metal impacted soils or acid mine drainages.

### Measuring ecological diversity and evaluating the importance of saturation and replicate sampling

In order to generate accurate estimates of diversity, our sampling scheme consisted of a replicated sampling design and large area of coverage of the stations [21]. We measured both alpha and beta diversity. **Alpha diversity** provides assessments of microbial richness in a particular natural environment. Comparisons of alpha diversity are *univariate*, two samples could have the same species richness or evenness but not share any taxa. In contrast, **beta diversity** measures (dis)similarity among samples through the use of *multivariate* methods that compare samples based on taxon composition and relative abundance. It is important to note that taxonomic description is not required for assessment of either of these measures.

#### Alpha diversity

Each method for estimating richness and comparing alpha diversity between samples has advantages and drawbacks [16], [17], [22]. Rarefaction, a measure of alpha diversity that reflects sample coverage at a site, is a good comparative method of *observed* microbial richness among samples at the same sampling effort [16]. Statistical differences in rarefaction curves among RT sample replicates from this study emphasize the importance of replication when measuring ecological diversity. In bacterial assemblages, most taxa are rare [23] and therefore rarefaction curves continue to increase with sampling effort and rarely reach an asymptote unless diversity is very low or sampling is very thorough. In contrast to rarefaction, coverage-based non-parametric richness estimators estimate *overall* species richness and compare alpha diversity of communities provided they reach an asymptote [16]. In this study, non-parametric estimators Chao1 and ACE ranged between 152 and 461 estimated OTUs for all the sites considered (Table 1). Chao1 values leveled off in more samples than ACE and it reached saturation in nearly all sites and in at least one sample per site (Table 1). Representation of Chao1 against sampling effort showed that alpha diversity is not significantly different among sites (P<0.05) at the same sampling effort (Fig. 4). This is effectively true for all sites except AG1 and AG3 because they did not reach saturation (Table 1). The highest OTU richness is found in the less extreme sites, a result that coincides with DGGE analysis [2]. The estimated overall number of OTUs is low in the RT sites compared to other environments with less extreme characteristics like soils or sediments where non-parametric estimators might not perform well (cf. [17]). When dealing with highly diverse samples Hong *et al.* have suggested a new set of statistical approaches to calculate microbial richness from parametric models [22].

**Table 1:** Alpha diversity measurements at Río Tinto samples and sites.

Samples	Number of tags	Number of OTUs	ACE	Chao1 (95% CIs)
**AG1.2**	485	77	210*	197*(133–326)
**AG1.3**	62	18	38	80(29–394)
**AG2.1**	291	44	149	136*(82–285)
**AG2.2**	116	15	45	35(22–93)
**AG2.3**	719	43	153	170*(89–419)
**AG3.1**	704	63	140	159*(105–294)
**AG3.2**	478	76	205*	220*(145–382)
**AG3.3**	624	101	274	251*(181–388)
**BE1.1**	543	87	332*	354*(207–688)
**BE1.2**	308	37	86	80*(55–152)
**BE1.3**	59	17	29	43*(23–141)
**BE2.1**	719	52	196*	148*(91–294)
**BE2.3**	462	68	333*	252*(151–486)
**BE3.1**	376	53	153	152*(95–298)
**BE3.3**	760	43	132	112*(70–232)
**OR1.1**	672	103	373	356(231–610)
**OR1.2**	334	57	115*	119(91–177)
**OR2.1**	551	72	235*	201*(135–341)
**OR2.2**	344	40	96	160(80–438)
**OR2.3**	451	76	219	202(138–345)
**OR3.1**	776	90	383	308(200–530)
**OR3.3**	695	51	123*	116*(86–175)
**Sites**				
**AG1**	547	83	231	211(141–363)
**AG2**	1126	81	399	268*(164–500)
**AG3**	1806	167	401	432(315–642)
**BE1**	879	112	461*	312*(216–496)
**BE2**	910	99	275*	277*(186–462)
**BE3**	1136	78	217*	152*(112–237)
**OR1**	1006	129	370*	310*(227–462)
**OR2**	1246	130	346	328*(234–505)
**OR3**	1471	110	222*	254*(184–391)

Number of tags, number of OTUs, and ACE and Chao1 non-parametric richness estimators arranged for each sample (top) and for pooled samples by site (bottom). The asterisk indicates estimators that plateau at a given number of OTUs.

#### Beta diversity

**Figure 5:**
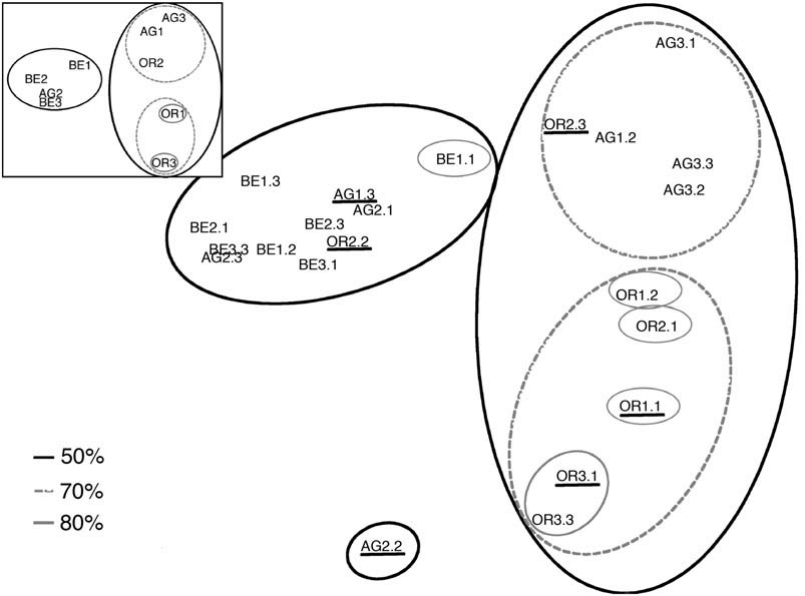
Beta diversity among Río Tinto samples and sites. Non-Metric Multi-dimensional Scaling plot of Morisita-Horn beta diversity indices among the different RT samples and sites (inset). Superimposed circles represent UPGMA clusters of samples (or sites) at similarity values of 50, 70 and 85%. Underlined samples represent samples wherein ACE and Chao1 richness estimators do not level-off (Table 1). For sample names see Materials and Methods.

To evaluate relationships among samples based on shared OTU relative abundance, we present results using the Morisita-Horn pairwise similarity coefficient. This index is widely used because it is less influenced by species richness and sample size than other (dis)similarity measures of quantitative data [16] and showed the best agreement between all methods employed to compare beta diversity in RT samples (see Materials & Methods). Non-metric Multi-Dimensional Scaling (MDS) ordination in conjunction with clustering analysis with the Unweighted Pair Group Method with Arithmetic mean (UPGMA) and ANalysis Of SIMilarities (ANOSIM) indicated a high similarity between Berrocal (BE) and AG2 samples (Fig. 5). Two other groups emerged from these analyses: one group included AG1 and AG3 samples and the other group Origin (OR) samples that further split at 85% similarity. Because assemblages vary in composition over space and time for stochastic reasons, sampling replication as well as saturation of alpha diversity help to capture the randomness of OTU recovery in microbial communities providing a more accurate estimate of beta diversity. For instance, OR2 samples were spread out in the 2-D plot (Fig. 5). This indicated poor replication in OTU composition of these samples, which is in agreement with the high OR2 site alpha diversity (Fig. 4). Only through replication do we obtain a better representation of the metapopulation at this site (Fig. 5 inset and see next section). Furthermore, samples that displayed unsaturated non-parametric alpha richness (Fig. 4) did not plot in the same position as when pooled by site (compare MDS plot of Fig. 5 with Fig. 5 inset) nor when comparing their distribution using environmental variables (see next section).

### Linking community attributes to environmental variables

**Figure 6:**
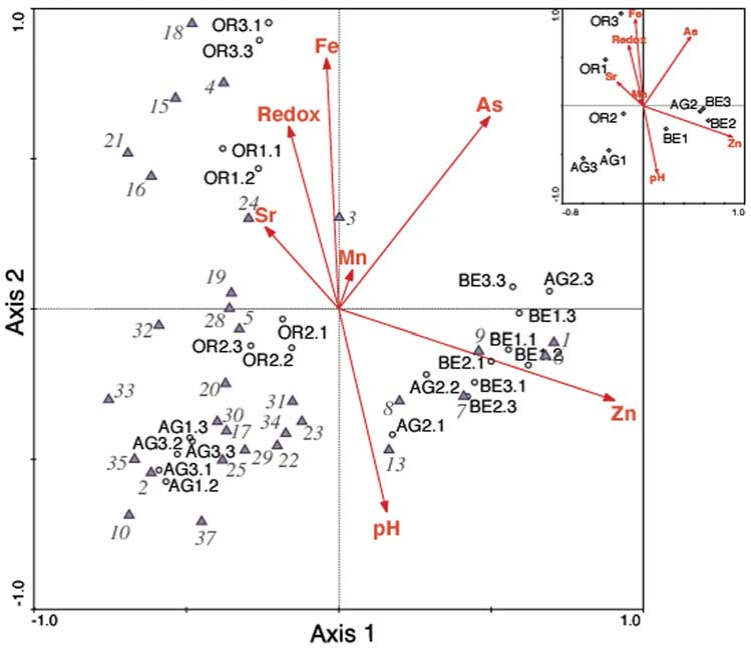
CCA biplot of the SARST-V6 dataset with relevant environmental variables at Río Tinto samples and sites. Superimposed canonical correspondence analysis (CCA) biplots of RT samples and SARST-V6 OTUs at the 99% similarity cut-off value displaying 68% of the variance of the OTUs with respect to the environmental variables. The inset represents the CCA biplot when pooling samples by site. The canonical eigenvalues for axes 1–4 of the sample analysis are 0.367, 0.272, 0.112, and 0.062 respectively. Environmental variables are indicated by arrows that point in the direction of increasing values of each variable. The coordinates of the arrowheads indicate the degree of correlation with the axes. Samples and sites are represented by black circles. For sample names see Materials and Methods. OTUs with total abundances higher than 10 RSTs are represented by grey triangles. To avoid overcrowding of points only one OTU per strain is plotted. The relative frequency of OTUs in samples can be determined using the biplot rule. To do this, drop a perpendicular from each sample onto a line through the OTU and the origin. Samples projecting on the line in the direction towards the OTU and beyond it are predicted to have a higher relative frequency of that OTU than samples projecting onto the line in the opposite direction. Interpretation of environmental arrows with respect to sites, OTUs and other environmental variables follows the same rule. OTU numbers correspond to: (1, 12, 14, 36) = *Acidithiobacillus* sp. SS5; (2, 11) = Uncultured bacterial clone MPKCSC9; (3) = *Acidithiobacillus* sp. SK5; (4) = *Leptospirillum ferrooxidans* P3a; (5, 26) = *L. ferrooxidans* Parys; (6) = *Acidithiobacillus* sp. B9; (7) = *L. ferrooxidans* Sy; (8) = *Thermicanus aegyptius*; (9) = *Acidiphilium* sp. Pk46; (10) = Eubacterium clones TRA5-3 and MeBr10; (13) = Uncultured bacterium BA18; (15) = *F. acidiphilium*; (16) = Bacterium clone 015C-C11; (17) = Actinomycetales clone TM167; (18) = *Leptospirillum* sp. strain DSM 2391; (19) = *Thermicanus aegyptius*; (20) = Bacterium Ellin5017; (21) = *Pseudomonas* sp. B35; (22) = *Nostoc* sp. PCC 9231; (23) = *Acidiphilium* sp. CCP3; (24) = Uncultured bacterium clone RCP2-12; (25) = Uncultured actinobacterium clone BPM2_A01; (27) = *Acidithiobacillus* sp. SK5; (28) = *Acidobacteria* clone BPC3_E10; (29) = Uncultured bacterium clone 300A-B12; (30) = Bacterium Ellin5114; (31) = *Corynebacterium* sp. S18-03; (32) = Uncultured bacterium clone RCP1-34; (33) = Uncultured bacterium clone RH1-L2; (34) = Uncultured bacterium clone RH1-i3; (35) = Uncultured bacterium clone RCP2-16; (37) = Uncultured actinobacterium clone BPM3_G08.

Amils *et al.* [24] proposed a geomicrobiological model for the RT controlled by iron and based on the geochemistry and the metabolism of the most abundant bacteria and archaea. The major non-photosynthetic primary producers *A. ferrooxidans* and *L. ferrooxidans* obtain their energy from pyrite (FeS_2_) and the oxidized metabolites can in turn be mineralized by heterotrophic microbes like *Acidiphilium* spp., *Ferromicrobium* or sulfate reducers. Ferric iron buffers the pH at or below pH 3. In our study, of the 22 environmental parameters measured, seven best explained the variation in the data (As, Fe, Mn, Sr, Zn, pH, and redox) (see Material & Methods). We used these variables to perform Canonical Correspondence Analysis (CCA) using OTUs at a 99% similarity cut-off with both samples and sites. The CCA plot for samples, sites or OTUs with respect to environmental variables showed a strong correlation of the canonical axes with the variables chosen (Fig. 6). Monte Carlo permutation tests for the first and all axes for samples and sites were highly significant (P = 0.002) indicating that these environmental parameters are important in explaining community diversity. For instance, AG2 is more similar in geochemistry and relative abundance of OTUs to BE (∼30 km away) than to AG1 and AG3, only meters away (Fig. 1). OTUs that plotted near BE and AG2 sites may therefore be better adapted to relatively higher concentrations of Zn and lower concentrations of As than OTUs with a higher relative abundance at other sites (Fig. 1 and 5). Furthermore, we observed that several OTUs had exactly the same match in GenBank and occupied the same position in the ordination plot (Fig. 6). We infer they are members of the same subspecific unit or ecotype that is better adapted to particular environmental characteristics.

### Implications for microbial community dynamics and biogeography

Stochastic vs. deterministic community assembly hypotheses are being tested in parallel to explain the distributional patterns of organisms in natural environments [25], [26]. In microbiology, the debate over the causes of niche apportionment started early in the nineteenth century. Baas Becking pointed towards a deterministic composition of the microbial communities with the hypothesis of “everything is everywhere, but, the environment selects” to explain his recurring observation of resuscitating microbial forms in enrichment cultures. This idea has generated much debate in recent years [27]. Hubbell's neutral theory of biodiversity and biogeography [28] examines the consequences of assuming a per capita ecological equivalence of trophically similar individuals of all sympatric species in a given community when shaped by ecological drift, random migration and random speciation. He concludes that these mechanisms decouple niche differentiation from control of species richness and relative species abundance in ecological communities. Sloan *et al.* [26] corroborate that immigration and chance are important processes shaping microbial communities demonstrating that stochastic neutral community models can describe the assemblage patterns of microorganisms. If we equate immigration with dispersal in the microbial world, dispersal and mutation are important processes driving bacterial population diversity patterns in the RT. Regarding mutation, we found that the most abundant OTUs are generally composed of a unique RST with the highest numbers of tags characterized by exact matches to sequences in GenBank (100% if it is a known species), and a few other unique RSTs with lower numbers of tags and correspondingly lower matches to sequences in GenBank. This pattern of within-OTU microdiversity cannot be explained by standard Taq error rates [12] alone and is best explained by high mutation rates in bacterial populations not yet being purged by selection. Genetic variation from mutation is an important process that might play a significant role in the population dynamics of asexual organisms [29]–[31]. In the long term, the ecosystem as a whole benefits from high biodiversity levels as it assures a good response to environmental variation. Yachi and Loreau [32] have referred to this as the “insurance effect”. With respect to dispersal, Hubbell's neutral model predicts species abundances to follow a log series distribution when immigration is unlimited if point mutation is the dominant form of speciation [28]; all RT samples from this study follow this model of species abundance when singletons are eliminated (p>0.05). Departure from the log series distribution at larger sampling efforts in RT samples might be explained by the high dispersal rate typical of bacteria (cf. [33]), which would make the tail of less abundant OTUs longer than expected for migration rates typical of macroorganisms. These less abundant taxa constitute allochthonous microbiota that arrive by dispersal and if they survive in RT extreme conditions they do so by competing for the leftovers from the dominant ecotypes. Our data agree with bacterial assemblages composed of “core” taxa and a “seed bank” [23]. The first are the most abundant and active organisms using relevant chemical elements as electron donors or acceptors and therefore adapted to fine changes in those elements in the system. The second are the “occasional taxa” derived from mutation and migration that might constitute a reservoir of diversity to respond to environmental changes (cf. [34]). For instance, the high abundance of the OTU that matches uncultured bacterial clones MPKCSC9 and TrefC11 in AG3 and AG1 sites (see above) contrasts with its low prevalence in AG2 (45 tags of 989 tags sequenced).

On the other hand, the presence of endemic ecotypes that correlate with particular environmental factors seem to contradict Baas Becking's ideas on global bacterial distribution [35], [36]. Our findings show that these ideas are not contradictory. A large number of OTUs match at 100% similarity those in geographically distant environments with similar physico-chemical characteristics (see above). Another interesting case is an OTU that had a 100% match to a sequence from a symbiont originally described from an amoeba in Iron Mountain (California) *Candidatus captivus acidiprotistae* [37]. This suggests a global distribution of its eukaryotic host via adaptation to low pH, and high Fe, As and Sr and Mn environments. Because we are dealing with a highly variable region of the small-subunit rRNA gene confirmed by the high mutation rate within each OTU, the presence of these highly similar sequences across the globe can only be explained if they are part of the same genetic pool. Coincidentally, the dominance of best competitors in a given environment is predicted by simulation when dispersal is not limited [38]. Thus, our results favor a scenario in which high immigration rates allow the *global* dispersion of ecotypes better adapted to certain environmental conditions, which prevail over less adapted units that emerge *locally*. Pommier *et al.* (2007) and Ramette *et al.* (2007) have suggested a similar pattern of global deterministic ecotype adaptation [39], [40]. Whether we consider this cosmopolitanism of ecotypes or local adaptation at a global scale is a question of lexical taste. Dispersal rather than niche differentiation is therefore the process eventually responsible for the observed deterministic pattern of most abundant members of the communities under this hypothesis reconciling neutral versus deterministic models of microbial community assemblage.

### Perspectives

Seasonal sampling that integrates bacterial, archaeal and eukaryal components of the microbial community is the necessary next step to understanding whether interaction of all trophic levels in the RT confirm or reject our scenario of the global distribution of adapted ecotypes.

## Materials and Methods

### Sampling sites, sample collection and DNA extraction

Our study included three stations in the RT that have distinct physico-chemical parameters and biology [2], [3], [41]; 1) the river's Origin (OR), 2) Anabel's Garden (AG) and 3) Berrocal (BE) (Fig. 1). At the OR station (N 37° 43.32′×W 6° 33.06′) we sampled three sites a few meters apart including OR3 that has some of the most extreme conditions along the river. The AG station (N 37° 43.49′×W 6° 33.62′) contains abundant and distinct biofilms. AG sampling sites are in a small stream and in a small ephemeral pool fed by seeps along the stream bank. The geochemical characteristics change over a relatively small spatial scale at AG. Higher water flow at BE station (N 37°35.58′×W 6° 33.04′) results in a well-mixed water column resulting in our most homogenous station. In October 2002, we sampled surface water in triplicate from three different sites at each of the three stations. We designated our samples using the following naming convention: Station abbreviation, site number, sample replicate number e.g. AG1.2 is the second replicate sample from site 1 at AG station. We rinsed 4 L plastic buckets three times with water from each site immediately before each replicate sample collection. We filtered 1–2 L from each sample by hand on site through 0.22 µm Sterivex filters (Millipore, Billerica, MA USA) and post-washed filters with 2 mL sterile acid water (pH 1.8).

We added Cell Lysis Solution from the Puregene® DNA extraction kit (Gentra Systems, Inc, Minneapolis, MI USA) directly to the sterivex filter using a 3cc. syringe, sealed the filter, and placed it into a liquid nitrogen dry shipper (Model SC14/2V, Custom BioGenic Systems, Shelby Township, MI). We extracted total DNA within one week of collection using the Puregene Bacteria DNA purification procedure with the following modifications. We added lysozyme (67 µL of 50 mg/mL solution) and proteinase K (10 µL at 20 mg/mL) consecutively directly to the sterivex filters and incubated these enzymes with agitation as indicated in the protocol. We then transferred incubated samples to three 2-mL microfuge tubes to proceed with the protein precipitation step. Nucleic acid precipitation occurred in 1 volume of isopropanol. Finally we resuspended DNA pellets in 30 µL Puregene© DNA Hydration Solution per sample and stored them at −20°C until further processing.

### Physicochemical measurements

For each sample both filtered and unfiltered 15 mL water aliquots were analyzed using Total Reflection X-ray Fluorescence (TXRF) at the Universidad Autónoma de Madrid (UAM Scientific Service, Spain) to determine the concentration of 22 chemical elements in the water samples examined. Given filtered vs. unfiltered geochemistries were not significantly different, we report results with unfiltered samples.

We measured redox potential and pH (using a Crison 506 pH/Eh meter) and conductivity (using a Orion-122 conductivity-meter) at the time of water collection from 15 mL aliquots. Oxygen concentration and water temperature were measured using an Orion-810 oxymeter *in situ* in the river at the time of water collection. These two parameters varied with time of day as the sun rose and heated the river so they were not included in our analyses.

### SARST-V6 amplification, sequencing, sequence analysis and Operational Taxonomic Unit (OTU) determination

SARST-V6 produces sequences of large concatemers of PCR-amplified ribosomal sequence tags (RSTs) from homologous V6 hypervariable regions. We performed amplification and purification of the V6 region of bacteria following [7] except that we used Accuprime™ Supermix (Invitrogen Inc., Carlsbad CA, USA) at a later phase of this project. PCR products were then ligated into concatemers, cloned and sequenced as previously described. A single sequence product contains information of multiple bacteria present in the DNA sample in the form of RSTs. The pipeline for SARST-V6 sequence analysis [42] parses concatemers into single RSTs, purges artifacts and pools RSTs into unique tag sequences. A combination of BLAST against the GenBank database (http://www.ncbi.nlm.nih.gov/GeneBank) and RDPQuery [43] against the RDPII database [44] guided taxonomic assignments of tags. A quality control step served to remove tags that hit non-ribosomal sequences, phage, virus, plasmid, chloroplast or vector sequences in GenBank. We then imported unique RSTs into ARB [45] along with the sequences of top GenBank and RDPII matches not already in ARB to generate a multiple sequence alignment used to pare-down tags that violated secondary structure in the V6 stem. The Clusterer program version 1.1.20060314 [46] served to group aligned sequences into OTUs. Because average and single linkage clustering algorithms are considered to be less conservative and more dependent on sampling intensity [47], we used the complete linkage algorithm with default parameters, except that we collapsed subsequent gaps to avoid overestimating distances from the rapidly diverging V6 region. We employed customized Perl scripts to construct abundance matrices accounting for the number of unique tag sequences per sample for each particular OTU and for each BLAST top hit GenBank gi number. Names of OTUs follow the first BLAST hit that match the most abundant RST of the cluster, regardless of the OTU's identity to other hits.

### Ecological diversity measurements

#### Alpha or inventory diversity

We first compared diversity between samples by representing relative abundances of OTUs in a rank/abundance plot. We then tested whether the data fit one of four statistical models of species abundance distributions: the geometric series, log normal, log series, or broken stick models. We transformed total abundance data into 0/1 matrices as input into the program EstimateS [48] to compute rarefaction curves, non-parametric richness estimators and several indices of alpha diversity.

#### Beta or differentiation diversity

We also used EstimateS to calculate Morisita-Horn, and the newly developed Chao-Jaccard and Chao-Sørensen abundance based beta similarity estimators [16], [49]. We further calculated Bray-Curtis similarities as similarity coefficients normalized by sample size using the PRIMER-E Ltd [50] software package. This software was used to perform non-metric multi-dimensional scaling (MDS) in conjunction with clustering analysis with the Unweighted Pair Group Method with Arithmetic mean (UPGMA). MDS was performed with 100 restarts at different random positions of samples to avoid local minima. To test the null hypothesis that there were no differences in community composition among sites we used ANalysis Of SIMilarities (ANOSIM) with the software PRIMER-E. ANOSIM is a simple non-parametric test better than the classical multivariate analysis of variance (MANOVA) [51] for this purpose [50]. It is based on the calculation of the R statistic over the rank similarities between samples, whose values can lie between −1 and 1. ANOSIM uses a Mantel permutation procedure combined with a randomization approach to generate significance levels (Monte Carlo tests).

We can consider our samples as real (independent) replicates of the studied sites because repetitive sampling was done independently from one sample to the next and the biological system under consideration is dynamic (the river water flows so the actual sampling space will never be the same from one moment to the next). This assumption not only validates ANOSIM analysis [50] but also allowed us to perform CCA with samples separately as replicates of the particular physico-chemical characteristics of a site to determine how samples behaved independently, and also because a larger number of samples allows for testing a larger number of environmental variables in constrained ordination. Although this is not always the case and careful consideration to this matter is necessary in microbial studies, this is the maximum level of replication that can be achieved when trying to explain beta diversity through environmental variables in natural environments (cf. [16]).

### Using environmental data to explain diversity data: Canonical Correspondence Analysis (CCA)

We used CCA as a constrained ordination direct gradient analysis method to relate RSTs grouped into OTUs to the environmental variables measured. We used CANOCO 4.5 [52] to perform CCA with scaling focused on inter-sample distances for the sample vs. environmental variable biplot and inter-species distances for the species vs. environmental variable biplot. These biplots were then superimposed. When performing constrained ordination it is important to limit the number of explanatory (environmental) variables to avoid exceeding the number of samples, otherwise the analysis becomes unconstrained and no different from indirect gradient analysis techniques such as DCA [52]. To perform CCA we used a combination of CANOCO's manual forward selection feature, Pearson correlation, and knowledge of the ecology of the river to select the environmental variables that could serve as proxies of others. To statistically evaluate the significance of the first canonical axis and of all canonical axes together, we used the Monte Carlo permutation full model test (whenever possible) or reduced model test with 199 unrestricted permutations. The program CANODRAW within the CANOCO package helped to visualize the resulting biplots.

## Supporting Information

Text S1 Environmental tag sequencing methods facilitate comprehensive microbial ecology and biogeography studies.
(0.05 MB DOC)

## References

[1] Fernández-Remolar D, Gómez-Elvira J, Sebastian E, Martín J, Manfredi JA (2004). The Tinto River, an extreme acidic environment under control of iron, as an analog of the Terra Meridiani hematite site of Mars.. Planet Space Sci.

[2] González-Toril E, Llobet-Brossa E, Casamayor EO, Amann R, Amils R (2003). Microbial ecology of an extreme acidic environment, the Tinto River.. Appl Environ Microbiol.

[3] Amaral Zettler LA, Gomez F, Zettler ER, Keenan BG, Amils R (2002). Eukaryotic diversity in Spain's River of Fire.. Nature.

[4] Aguilera A, Manrubia SC, Gomez F, Rodriguez N, Amils R (2006). Eukaryotic community distribution and its relationship to water physicochemical parameters in an extreme acidic environment, Rio Tinto (Southwestern Spain).. Appl Environ Microbiol.

[5] Karavaiko GI, Turova TP, Kondrat'eva TF, Lysenko AM, Kolganova TV (2003). Phylogenetic heterogeneity of the species Acidithiobacillus ferrooxidans.. Int J Syst Evol Microbiol.

[6] Green BD, Keller M (2006). Capturing the uncultivated majority.. Curr Opin Biotechnol.

[7] Kysela DT, Palacios C, Sogin ML (2005). Serial analysis of V6 ribosomal sequence tags (SARST-V6): a method for efficient, high-throughput analysis of microbial community composition.. Environ Microbiol.

[8] Stackebrandt E, Goebel B (1994). Taxonomic note: A place for DNA-DNA reassociation and 16S rRNA sequence analysis in the present species definition in bacteriology.. INt J Syst Bacteriol.

[9] Cohan FM (2006). Towards a conceptual and operational union of bacterial systematics, ecology, and evolution.. Philos Trans R Soc Lond B Biol Sci.

[10] Polz M, Hunt D, Preheim S, Weinreich D (2006). Patterns and mechanisms of genetic and phenotypic differentiation in marine microbes.. Philos Trans R Soc Lond B Biol Sci.

[11] Klepac-Ceraj V, Bahr M, Crump BC, Teske AP, Hobbie JE (2004). High overall diversity and dominance of microdiverse relationships in salt marsh sulphate-reducing bacteria.. Environ Microbiol.

[12] Acinas SG, Klepac-Ceraj V, Hunt DE, Pharino C, Ceraj I (2004). Fine-scale phylogenetic architecture of a complex bacterial community.. Nature.

[13] Koeppel A, Perry EB, Sikorski J, Krizanc D, Warner A (2008). Identifying the fundamental units of bacterial diversity: A paradigm shift to incorporate ecology into bacterial systematics.. Proc Natl Acad Sci U S A.

[14] Thompson JR, Pacocha S, Pharino C, Klepac-Ceraj V, Hunt DE (2005). Genotypic diversity within a natural coastal bacterioplankton population.. Science.

[15] Cilia V, Lafay B, Christen R (1996). Sequence heterogeneities among 16S ribosomal RNA sequences, and their effect on phylogenetic analyses at the species level.. Mol Biol Evol.

[16] Magurran AE (2004). Measuring biological diversity.

[17] Hughes JB, Hellmann JJ, Ricketts TH, Bohannan BJ (2001). Counting the uncountable: statistical approaches to estimating microbial diversity.. Appl Environ Microbiol.

[18] Hallberg KB, Coupland K, Kimura S, Johnson DB (2006). Macroscopic streamer growths in acidic, metal-rich mine waters in North Wales consist of novel and remarkably simple bacterial communities.. Appl Environ Microbiol.

[19] Slayman C (1985). Proton chemistry and the ubiquity of proton pumps.. BioScience.

[20] Sogin ML, Morrison HG, Huber JA, Welch DM, Huse SM (2006). Microbial diversity in the deep sea and the underexplored “rare biosphere”.. Proc Natl Acad Sci U S A.

[21] Dobyns JR (1997). Effects of sampling intensity on the collection of spider (Araneae) Species and the estimation of species richness.. Environ Entomol.

[22] Hong S-H, Bunge J, Jeon S-O, Epstein SS (2006). Predicting microbial species richness.. Proc Natl Acad Sci U S A.

[23] Pedros-Alio C (2006). Marine microbial diversity: can it be determined?. Trends Microbiol.

[24] Amils R, Gonzalez-Toril E, Fernández-Remolar D, Gomez F, Aguilera A (2007). Extreme environments as Mars terrestrial analogs: The Rio Tinto case.. Planetary and Space Science.

[25] McGill BJ, Maurer BA, Weiser MD (2006). Empirical evaluation of neutral theory.. Ecology.

[26] Sloan WT, Lunn M, Woodcock S, Head IM, Nee S (2006). Quantifying the roles of immigration and chance in shaping prokaryote community structure.. Environ Microbiol.

[27] de Wit R, Bouvier T (2006). ‘Everything is everywhere, but, the environment selects’; what did Baas Becking and Beijerinck really say?. Environ Microbiol.

[28] Hubbell SP (2001). The Unified Neutral Theory of Biodiversity and Biogeography.

[29] Boles BR, Thoendel M, Singh PK (2004). From the Cover: Self-generated diversity produces “insurance effects” in biofilm communities.. Proceedings of the National Academy of Sciences.

[30] de Visser JAGM, Rozen DE (2006). Clonal Interference and the Periodic Selection of New Beneficial Mutations in Escherichia coli.. Genetics.

[31] Yooseph S, Sutton G, Rusch DB, Halpern AL, Williamson SJ (2007). The Sorcerer II Global Ocean Sampling expedition: expanding the universe of protein families.. PLoS Biol.

[32] Yachi S, Loreau M (1999). Biodiversity and ecosystem productivity in a fluctuating environment: the insurance hypothesis.. Proc Natl Acad Sci U S A.

[33] Magurran AE, Henderson PA (2003). Explaining the excess of rare species in natural species abundance distributions.. Nature.

[34] Falkowski PG, Godfrey LV (2008). Electrons, life and the evolution of Earth's oxygen cycle.. Philos Trans R Soc Lond B Biol Sci.

[35] Whitaker RJ, Grogan DW, Taylor JW (2003). Geographic barriers isolate endemic populations of hyperthermophilic archaea.. Science.

[36] Pommier T, Pinhassi J, Hagström Å (2005). Biogeographic analysis of ribosomal RNA clusters from marine bacterioplankton.. Aquat Microb Ecol.

[37] Baker BJ, Hugenholtz P, Dawson SC, Banfield JF (2003). Extremely acidophilic protists from acid mine drainage host Rickettsiales-lineage endosymbionts that have intervening sequences in their 16S rRNA genes.. Appl Environ Microbiol.

[38] Hurtt GC, Pacala SW (1995). The consequences of recruitment limitation: Reconciling chance, history, and competitive differnces between plants.. J Theor Biol.

[39] Pommier T, Canback B, Riemann L, Bostrom KH, Simu K (2007). Global patterns of diversity and community structure in marine bacterioplankton.. Mol Ecol.

[40] Ramette A, Tiedje JM (2007). Biogeography: an emerging cornerstone for understanding prokaryotic diversity, ecology, and evolution.. Microb Ecol.

[41] Lopez-Archilla AI, Marin I, Amils R (2001). Microbial Community Composition and Ecology of an Acidic Aquatic Environment: The Tinto River, Spain.. Microbial Ecology.

[42] Palacios C, Olsson B, Lebaron P, Sogin ML (2006). New high-throughput biotechnologies for sampling the microbial ecological diversity of the oceans: the informatics challenge..

[43] Dyszynski G, Sheldon WM RDPquery: A Java program from the Sapelo Program Microbial Observatory for automatic classification of bacterial 16S rRNA sequences based on Ribosomal Database Project taxonomy and Smith-Waterman alignment.. http://simo.marsci.uga.edu/public_db/rdp_query.htm.

[44] Cole JR, Chai B, Farris R, Wang Q, Kulam SA (2005). The Ribosomal Database Project (RDP-II): sequences and tools for high-throughput rRNA analysis.. Nucleic Acids Res.

[45] Ludwig W, Strunk O, Westram R, Richter L, Meier H (2004). ARB: a software environment for sequence data.. Nucleic Acids Res.

[46] Klepac-Ceraj V, Ceraj I, Polz MF (2006). Clusterer: extendable java application for sequence grouping and cluster analyses.. Online J Bioinformatics.

[47] Schloss PD, Handelsman J (2005). Introducing DOTUR, a Computer Program for Defining Operational Taxonomic Units and Estimating Species Richness.. Appl Environ Microbiol.

[48] Colwell RK (2005). EstimateS: Statistical estimation of species richness and shared species from samples.. http://purl.oclc.org/estimates.

[49] Chao A, Chazdon RL, Colwell RK, Shen T-J (2005). A new statistical approach for assessing compositional similarity based on incidence and abundance data.. Ecology Letters.

[50] Clarke KR, Warwick RW (2001). Change in marine communities: an approach to statistical analysis and interpretation, 2nd edition.

[51] Legendre P, Legendre L (1998). Numerical ecology.

[52] ter Braak CJF, Šmilauer P (2002). CANOCO Reference Manual and CanoDraw for Windows User's Guide: Software for Canonical Community Ordination (version 4.5).

