# Supplementary material for: Contrasting Microbial Community Assembly Hypotheses: A Reconciling Tale from the Río Tinto
Source: PLoS One. 2008 Dec 4;3(12):e3853. doi: 10.1371/journal.pone.0003853 (PMC2587236; doi:10.1371/journal.pone.0003853)
Supplement: Text S1 — Environmental tag sequencing methods facilitate comprehensive microbial ecology and biogeography studies. (0.05 MB DOC) [file pone.0003853.s001.doc]

**SUPPORTING INFORMATION**

# Text S1

## Environmental tag sequencing methods facilitate comprehensive microbial ecology and biogeography studies.

The abundance of rDNA sequences present in public databases allows implementation of environmental tag sequencing methods [1] like SARST [2-5]. Because they are based on sequencing, these methods are better suited to high-throughput data recovery when compared to fingerprinting techniques like TRFLP, DGGE or ARISA in which additional sequencing is often required for full description of the microbial communities. Estimating relative abundances is essential when characterizing both alpha and beta diversity [6,7] and for this reason, environmental tag libraries have advantages over methods like FISH, standard rDNA libraries or metagenomics surveys [8]. Because tag methods target smaller size molecules, they allow for more rigorous nucleic acid extractions, reduce amplification bias, and likely minimize the formation of chimeras [4]. Cloning bias is eliminated through random assemblage of tags into concatemers prior to ligation, but PCR primer bias can only be avoided through improved design of oligonucleotides from reliable databases. SARST-V6 primers fall within a frequently sequenced region of the rDNA; our primers match at least 81% of currently known rDNA sequences from bacteria (estimated using probematch tool of the RDP II database with a 2 base pair mismatch in March 2008). Also rrn operon heterogeneity should not mislead identification of phylotypes when dealing with short and highly evolving regions such as those amplified with tag methods (cf. [9,10]). In summary, sequencing of ribosomal sequence tags through concatemerization [4] or through new pyrosequencing technology [11] offers high throughput of potentially unbiased, first generation information of the taxonomic affinities and relative abundances of organisms directly from natural samples. SARST-V6 in particular, provides an alternative to laboratories with no access to expensive 454 pyrosequencing. These approaches will facilitate comprehensive microbial ecology studies that can elucidate evolutionary and ecological processes underlying microbial community structures.

## References

1. Green BD, Keller M (2006) Capturing the uncultivated majority. Curr Opin Biotechnol 17: 236-240.

2. Neufeld JD, Yu Z, Lam W, Mohn WW (2004) Serial analysis of ribosomal sequence tags (SARST): a new high-throughput method for profiling complex microbial communities. Environ Microbiol 6: 131-144.

3. Neufeld JD, Mohn WW (2005) Unexpectedly high bacterial diversity in arctic tundra relative to boreal forest soils, revealed by serial analysis of ribosomal sequence tags. Appl Environ Microbiol 71: 5710-5718.

4. Kysela DT, Palacios C, Sogin ML (2005) Serial analysis of V6 ribosomal sequence tags (SARST-V6): a method for efficient, high-throughput analysis of microbial community composition. Environ Microbiol 7: 356-364.

5. Yu Z, Yu M, Morrison M (2006) Improved serial analysis of V1 ribosomal sequence tags (SARST-V1) provides a rapid, comprehensive, sequence-based characterization of bacterial diversity and community composition. Environ Microbiol 8: 603-611.

6. Hughes JB, Hellmann JJ, Ricketts TH, Bohannan BJ (2001) Counting the uncountable: statistical approaches to estimating microbial diversity. Appl Environ Microbiol 67: 4399-4406.

7. Magurran AE (2004) Measuring biological diversity. Malden: Blackwell Science Ltd. 256 p.

8. Huber JA, Welch DB, Morrison HG, Huse SM, Neal PR, et al. (2007) Microbial population structures in the deep marine biosphere. Science 318: 97-100.

9. von Wintzingerode F, Gobel UB, Stackebrandt E (1997) Determination of microbial diversity in environmental samples: pitfalls of PCR-based rRNA analysis. FEMS Microbiol Rev 21: 213-229.

10. Case RJ, Boucher Y, Dahllof I, Holmstrom C, Doolittle WF, et al. (2007) Use of 16S rRNA and rpoB Genes as Molecular Markers for Microbial Ecology Studies. Appl Environ Microbiol 73: 278-288.

11. Sogin ML, Morrison HG, Huber JA, Welch DM, Huse SM, et al. (2006) Microbial diversity in the deep sea and the underexplored "rare biosphere". Proc Natl Acad Sci U S A 103: 12115-12120.
